# Prevalence and correlates of different smoking bans in homes and cars among smokers in six countries of the EUREST-PLUS ITC Europe Surveys

**DOI:** 10.18332/tid/94827

**Published:** 2019-01-31

**Authors:** Marcela Fu, Yolanda Castellano, Olena Tigova, Christina N. Kyriakos, Geoffrey T. Fong, Ute Mons, Witold A. Zatoński, Thomas K. Agar, Anne C. K. Quah, Antigona C. Trofor, Tibor Demjén, Krzysztof Przewoźniak, Yannis Tountas, Constantine I. Vardavas, Esteve Fernández, on behalf of the EUREST-PLUS consortium

**Affiliations:** 1 Catalan Institute of Oncology, Catalonia, Spain; 2 Bellvitge Biomedical Research Institute (IDIBELL), Catalonia, Spain; 3 University of Barcelona, Catalonia, Spain; 4 European Network on Smoking and Tobacco Prevention (ENSP), Brussels, Belgium; 5 University of Crete (UoC), Heraklion, Greece; 6 University of Waterloo (UW), Waterloo, Canada; 7 Ontario Institute for Cancer Research, Toronto, Canada; 8 Cancer Prevention Unit and WHO Collaborating Centre for Tobacco Control, German Cancer Research Centre (DKFZ), Heidelberg, Germany; 9 Health Promotion Foundation (HPF), Warsaw, Poland; 10 European Observatory of Health Inequalities, The President Stanisław Wojciechowski State University of Applied Sciences, Kalisz, Poland; 11 University of Medicine and Pharmacy ‘Grigore T. Popa’ Iasi, Iasi, Romania; 12 Aer Pur Romania, Bucharest, Romania; 13 Smoking or Health Hungarian Foundation (SHHF), Budapest, Hungary; 14 Maria Skłodowska-Curie Institute-Oncology Center, Warsaw, Poland; 15 National and Kapodistrian University of Athens (UoA), Athens, Greece

**Keywords:** voluntary smoke-free regulation, private settings, smokers, Europe, WHO FCTC

## Abstract

**INTRODUCTION:**

Second-hand smoke exposure has decreased in a number of countries due to widespread smoke-free legislation in public places, but exposure is still present in private settings like homes and cars. Our objective was to describe to what extent smokers implement smoking rules in these settings in six European Union (EU) Member States (MS).

**METHODS:**

A cross-sectional survey was conducted with a nationally representative sample of adult smokers from Germany, Greece, Hungary, Poland, Romania and Spain (ITC six European countries survey, part of the EUREST-PLUS Project). We analysed data from 6011 smokers regarding smoking rules in their homes and in cars with children (no rules, partial ban, total ban). We described the prevalence of smoking rules by EU MS and several sociodemographic and smoking characteristics using prevalence ratios (PR) and 95% confidence intervals (CI) derived from Poisson regression models. \

**RESULTS:**

In homes, 26.5% had a total smoking ban (from 13.1% in Spain to 35.5% in Hungary), 44.7% had a partial ban (from 41.3% in Spain to 49.9% in Greece), and 28.8% had no-smoking rules (from 20.2% in Romania to 45.6% in Spain). Prevalence of no-smoking rules in cars with children was 16.2% (from 11.2% in Germany to 20.4% in Spain). The correlates of not restricting smoking in homes and cars included: low education (PR=1.51; 95%CI: 1.20-1.90 and PR=1.55; 95%CI: 1.09-2.20), smoking >30 cigarettes daily (PR=1.53; 95%CI: 1.10-2.14 and PR=2.66; 95%CI: 1.40-5.05) and no attempts to quit ever (PR=1.18; 95%CI: 1.06-1.31 and PR=1.28; 95%CI: 1.06-1.54).

**CONCLUSIONS:**

Among smokers in six EU MS, no-smoking rules were more prevalent in homes than in cars with children. Whilst awareness about the health effects of exposure to tobacco smoke on children seemed to be high, more research is needed to better understand the factors that promote private smoke-free environments.

## INTRODUCTION

Second-hand tobacco smoke is one of the most widespread air pollutants in indoor environments^1^. Exposure to it has been linked to several health outcomes, including respiratory asthma, infections of the lower respiratory tract, otitis media and sudden infant death syndrome, as well as ischaemic heart disease and lung cancer in adults^1^. The World Health Organization (WHO) Framework Convention on Tobacco Control (FCTC), an international health treaty, promotes smoke-free environments to achieve effective protection from the hazards of secondhand tobacco smoke. Article 8 of the FCTC calls upon Parties to implement measures for protection from exposure to tobacco smoke in public places, including but not limited to indoor workplaces, public transportation, indoor public places, and other public places^2^. Guidelines for the implementation of Article 8 have been developed further to assist countries in the adoption and implementation of smoke-free measures, as well as in identifying the key elements of their smoke-free legislation^3^.

Ratification of the WHO FCTC is associated with the accelerated implementation of key demand-reduction measures, including smoke-free laws^4,5^, with evidence of significant dose-respondent decreases in smoking prevalence and highest-level implementation of these measures^6^. Since 2004, many countries have adopted complete national smoking bans in public places, which has resulted in benefits to both non-smokers (less second-hand smoke exposure) and smokers (tend to smoke less, have greater cessation success, and experience more confidence in their ability to quit)7. However, only 16% of the world’s population is covered by comprehensive smoke-free laws^7^. Furthermore, these policies at the national level have only been adopted within the public domain and do not usually apply to private settings, such as homes and cars, where exposure to second-hand tobacco smoke is still common^8,9^. Nevertheless, some studies indicate that smoke-free legislation has had unexpected effects in promoting private smoke-free settings^10,11^. It has been hypothesised that public policies affect a variety of psychosocial and behavioural variables^12^. We hypothesise that the implementation of robust smoke-free legislation in public places will lead to the subsequent cessation of smoking in public places; this will in a second stage lead to changes in psychological mediators (e.g. awareness of the health effects of second-hand tobacco smoke) and in a third stage to behavioural changes, such as the establishment of voluntary tobacco-free policies in private settings.

Little is known on the extent to which smokers implement smoke-free bans within their own private settings. Thus, the objective of this study was to assess existing smoking rules and their correlates in smokers’ private settings, such as homes and cars, in six European countries.

## METHODS

### Design

This study is part of the European Commission Horizon-2020 funded study entitled *European Regulatory Science on Tobacco: Policy implementation to reduce lung diseases* (EUREST-PLUSHCO-06-2015; https://eurestplus.eu/), with main objective to monitor and assess the impact of the ratification of the WHO FCTC at the European level, through the implementation of the Tobacco Products Directive (2014/40/EU)^13^. To achieve this goal, a prospective cohort study of approximately 6000 smokers began using representative samples of smokers in each of the following six European Union (EU) Member States (MS): Germany (n=1003), Greece (n=1000), Hungary (n=1000), Poland (n=1006), Romania (n=1001), and Spain (n=1001).

This cohort survey, the ITC six European countries survey (ITC 6E Survey), is part of the ongoing International Tobacco Control Project Policy Evaluation (ITC) Project (www.itcproject.org/), which aims at tracking and comparing the impact of national-level tobacco policies among representative samples of adult smokers in 29 countries. The methods used in the ITC 6E Survey are explained elsewhere^14^. Briefly, samples of adult (≥18 years old) current smokers (having smoked >100 cigarettes in their lifetime and having smoked at least once in the past 30 days) were recruited using probability sampling methods, representative of all geographical regions in each EU MS. Eligible households were randomly selected using a random-walk method, and a household was considered eligible if it included at least one eligible smoker. Where available, both one male and one female smoker were selected from each household using the last birthday method^15^. Baseline data (i.e. Wave 1 of the ITC 6E Survey) were collected over a month, in each EU MS, between June and September 2016. After informed consent was provided, a computer-assisted personal interview was conducted. Participants received remuneration as an incentive for their participation. The study protocol was approved by an ethics committee in each participating country and partnering institutions.

This report is a cross-sectional baseline analysis of data collected from Wave 1 on the degree to which smoking rules are implemented in private indoor settings, namely, in smokers’ homes and in their cars in the presence of children.

### Measures

#### Smoking rules in homes

Rules in homes were ascertained by the question: ‘Which of the following statements best describes smoking inside your home? I mean inside your house or dwelling and not on the balcony, terrace, or other outdoor areas’. The possible answers were: ‘smoking allowed anywhere inside the home’, ‘smoking allowed in some rooms inside the home’, ‘smoking never allowed anywhere inside the home’, and ‘smoking not allowed inside home except under special circumstances’. These possible answers were re-coded as ‘no rules’ (first possible answer), ‘partial ban’ (second and last possible answers), and ‘total ban’ (third possible answer).

#### Smoking rules in cars

Rules in cars were ascertained with the question: ‘What are the rules about smoking in your car or cars when there are children in the car?’. The possible answers were: ‘smoking never allowed in any car’, ‘smoking allowed sometimes or in some cars’, and ‘smoking allowed in all cars’. These possible answers were re-coded as ‘total ban’, ‘partial ban’ and ‘no rules’, respectively. Answers ‘do not have a car’ or ‘never have children in car’ were excluded from the analyses.

### Analysis

We describe the prevalence of different smoking rules (no rules, partial and total smoking ban) in homes and in cars of smokers by EU MS, by several sociodemographic (sex, age, educational level - a country-specific variable standardised and categorised as low, intermediate, and high -, partner’s smoking status, having children) and by smoking characteristics (cigarettes smoked per day - CPD, nicotine dependence measured by the Heaviness of Smoking Index^16^, and attempts to quit smoking). We also conducted pairwise comparisons of smoking rules (partial vs total ban; no rules vs total ban) according to all independent variables using prevalence ratios (PR) and their 95% confidence intervals (CI) derived from Poisson regression models with robust variance, adjusting for all independent variables. All the analyses incorporated the weights derived from the complex sampling design. We used Stata v.13 for all analyses.

## RESULTS

### Smoking rules at home

**Figure 1:**
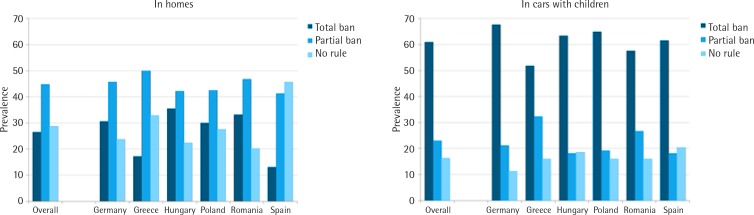
Prevalence of smoking rules in smokers’ homes and cars with children, 2016

Among all smokers across the six countries, 26.5% reported a total smoking ban in their home, with Hungary having the highest prevalence (35.5%) and Spain the lowest (13.1%); see Figure 1 and Supplementary Table 1. On the other hand, 28.8% of smokers across the six countries had no-smoking rules in their homes, varying from 20.2% in Romania to 45.6% in Spain. Overall, the prevalence of a total smoking ban in homes was similar in both sexes, but was significantly lower among participants aged 55 years or older and among those with lower educational level (Table 1). The prevalence of a total smoking ban in homes was higher among smokers with a non-smoker partner and among those with more children. Forty-two per cent of smokers who had children less than one year of age had a total smoking ban in their homes, varying from 11.4% in Spain to 56.6% in Romania (Supplementary Table 2). Additionally, there was a higher prevalence of total smoking bans among homes of smokers who reported to smoke less, were least nicotine dependent, and who had ever attempted to quit smoking (Table 1). Information for each EU MS is provided in the Supplementary Table 2.

**Table 1:** Prevalence of smoking rules in smokers’ homes, according to sociodemographic and smoking characteristics, 2016

	*Overall*		*Total ban*		*Partial ban*		*No rules*		
	*n*	*%^a^*	*n*	*%^b^*	*n*	*%^b^*	*n*	*%^b^*	*p^c^*
**Overall**	5967	100.0	1686	26.5	2661	44.7	1620	28.8	<0.001
**Sex**									<0.001
Men	3155	57.4	910	26.7	1328	42.6	917	30.7	
Women	2812	42.6	776	26.4	1333	47.6	703	26.0	
**Age (years)**									<0.001
18−24	507	10.1	161	28.8	188	36.8	158	34.4	
25−39	1757	31.5	553	29.6	782	45.3	422	25.1	
40−54	1992	34.1	534	25.7	957	46.5	501	27.8	
≥ 55	1711	24.4	438	22.9	734	44.8	539	32.3	
**Educational level**									<0.001
Low	2207	37.7	563	23.5	951	42.6	693	33.9	
Intermediate	3074	51.4	909	28.2	1382	45.3	783	26.5	
High	652	10.9	206	29.7	311	48.9	135	21.4	
**Smoker partner**									<0.001
Yes	2242	58.9	569	24.6	1088	47.9	585	27.5	
No	1629	41.1	614	35.9	737	45.8	278	18.3	
**Children**									<0.001
Yes	1958	33.6	702	34.6	929	47.2	327	18.2	
No	4001	66.4	982	22.5	1729	43.5	1290	34.0	
**Number of children**									<0.001
0	4001	66.4	982	22.5	1729	43.5	1290	34.0	
1	1000	17.1	352	34.3	493	49.5	155	16.2	
2	709	12.1	280	37.8	314	43.4	115	18.8	
≥3	249	4.4	70	27.6	122	48.3	57	24.1	
**Children’s age (years)^d^**									
<1	140	7.9	59	42.0	65	47.4	16	10.6	0.041
1−5	731	38.6	287	37.4	319	44.1	125	18.5	0.149
6−12	1029	53.2	360	32.9	505	48.9	164	18.2	0.247
13−17	760	37.4	255	33.1	356	46.0	149	20.9	0.119
**Cigarettes smoked per day**									<0.001
≤ 10	2063	33.2	764	35.9	892	43.2	407	20.9	
11−20	3059	52.8	771	23.5	1405	45.7	883	30.8	
21−30	523	8.9	97	16.9	224	45.2	202	37.9	
>30	314	5.1	51	14.3	138	44.2	125	41.5	
**Nicotine dependence**									<0.001
Low	2341	40.0	802	33.5	1021	43.7	518	22.8	
Medium	2799	51.1	680	22.3	1277	45.4	842	32.3	
High	516	9.0	74	13.0	222	44.0	220	43.0	
**Attempts to quit smoking**									<0.001
Yes	3223	53.4	990	29.0	1439	45.3	794	25.7	
No	2741	46.6	695	23.6	1221	44.1	825	32.3	

a Weighted percentages per column.

b Weighted percentages per row.

c χ^2^ test.

d Multiple response.

**Table 2:** Comparison of smoking rules (partial ban vs total ban; no rules vs total ban) in smokers’ homes, according to independent variables, 2016

	*Partial ban vs Total ban*			*No rules vs Total ban*		
	*PR*	*95% CI*	*p^a^*	*PR*	*95% CI*	*p^a^*
**Country**						
Germany	1	-		1	-	
Greece	1.23	1.07−1.42	0.005	1.68	1.29−2.19	<0.001
Hungary	0.94	0.80−1.10	0.417	1.12	0.85−1.47	0.422
Poland	0.99	0.85−1.15	0.883	1.33	1.04−1.71	0.026
Romania	1.07	0.92−1.24	0.394	1.27	0.97−1.65	0.082
Spain	1.40	1.23−1.60	<0.001	2.38	1.89−2.98	<0.001
**Sex**						
Men	1	-		1	-	
Women	1.09	1.04−1.15	0.001	1.12	1.02−1.22	0.014
**Age (years)**						
18−24	1.04	0.87−1.24	0.692	1.15	0.90−1.47	0.259
25−39	1.04	0.94−1.15	0.445	1.01	0.88−1.15	0.898
40−54	1.04	0.95−1.14	0.356	0.96	0.85−1.09	0.558
≥55	1	-		1	-	
**Educational level**						
Low	1.05	0.94−1.18	0.370	1.51	1.20−1.90	0.001
Intermediate	1.00	0.90−1.12	0.995	1.21	0.97−1.51	0.097
High	1	-		1	-	
**Smoker partner**						
Yes	1.18	1.10−1.27	<0.001	1.49	1.33−1.68	<0.001
No	1	-		1	-	
**Children**						
Yes	1	-		1	-	
No	1.19	1.10−1.29	<0.001	1.64	1.44 −1.86	<0.001
**Cigarettes smoked per day**						
≤10	1	-		1	-	
11−20	1.22	1.12−1.34	<0.001	1.41	1.20−1.64	<0.001
21−30	1.22	1.06−1.42	0.007	1.52	1.22−1.90	<0.001
>30	1.18	0.94−1.48	0.147	1.53	1.10−2.14	0.013
**Nicotine dependence**						
Low	1	-		1	-	
Medium	1.03	0.94−1.13	0.486	1.17	1.01−1.36	0.038
High	1.15	0.96−1.39	0.128	1.33	1.01−1.76	0.044
**Attempts to quit smoking**						
Yes	1	-		1	-	
No	1.06	0.99−1.13	0.090	1.18	1.06 −1.31	0.002

Prevalence ratios (PR) are derived from Poisson regression models, adjusted for all independent variables.

a χ^2^ test.

We further assessed the association of smoking rules in homes (partial vs total ban; no rules vs total ban) with different independent variables, where prevalence ratios >1 indicate less smoking rules in homes (Table 2). Compared to smokers from Germany, the countries where smokers were more likely to have a partial smoking ban or no-smoking rules in their homes were Spain (PR=1.40, 95% CI: 1.23-1.60 for partial vs total smoking ban; PR=2.38, 95% CI: 1.89-2.98 for no-smoking rules vs total smoking ban) and Greece (PR=1.23, 95% CI: 1.07-1.42 for partial vs total smoking ban; PR=1.68, 95% CI: 1.29-2.19 for no-smoking rules vs total smoking ban). The variables significantly associated with not having smoking rules in homes were: female, low educational level, partner who smokes, no children, more CPD, more nicotine dependent, and never attempted to quit smoking (Table 2).

### Smoking rules in cars with children

Overall, 60.9% of smokers stated that they had a total smoking ban in their cars when children were present, 22.9% stated having a partial ban, and 16.2% stated having no-smoking rules at all. Smoking rules varied across EU MS, with Greece having the lowest prevalence for cars with a total smoking ban (51.8%) and Germany having the highest prevalence (67.7%); see Figure 1 and Supplementary Table 1. Analyses examining associations with other independent variables showed that cars with total smoking bans were more prevalent among female smokers, and this increased with age and with educational level.

The prevalence of total smoking bans in cars was also higher among smokers with a non-smoker partner, with more children, among those who smoked fewer CPD, with lower nicotine dependence, and with any ever attempt to quit smoking (Table 3). Overall, smokers consuming >30 CPD were the only group with a prevalence of a total smoking ban in cars of less than 50% (Table 3), varying from 32.6% in Greece to 59.0% in Spain. Information by country is provided in the Supplementary Table 3.

**Table 3:** Prevalence of smoking rules in smokers’ cars with children, according to sociodemographic and smoking characteristics, 2016

	*Overall*		*Total ban*		*Partial ban*		*No rules*		
	*n*	*%^a^*	*n*	*%^b^*	*n*	*%^b^*	*n*	*%^b^*	*p^c^*
**Overall**	3885	100	2409	60.9	839	22.9	637	16.2	<0.001
**Sex**									<0.001
Men	2060	57.4	1211	57.9	481	24.3	368	17.8	
Women	1825	42.6	1198	64.9	358	20.9	269	14.2	
**Age (years)**									<0.001
18−24	272	10.1	151	51.7	65	25.8	56	22.5	
25−39	1198	31.5	729	61.7	259	21.9	210	16.4	
40−54	1408	34.1	856	59.1	340	25.5	212	15.4	
≥ 55	1007	24.4	673	66.3	175	18.5	159	15.2	
**Educational level**									0.013
Low	1268	37.7	763	58.8	269	22.1	236	19.1	
Intermediate	2091	51.4	1300	61.2	447	22.9	344	15.9	
High	504	10.9	332	65.1	116	24.2	56	10.7	
**Smoker partner**									0.470
Yes	1611	58.9	993	60.9	351	22.8	267	16.3	
No	1196	41.1	780	63.6	238	21.5	178	14.9	
**Children**									0.354
Yes	1467	33.6	925	62.5	317	22.4	225	15.1	
No	2412	66.4	1482	60.0	519	23.0	411	17.0	
**Number of children**									0.047
0	2412	66.4	1482	60.0	519	23.0	411	17.0	
1	761	17.1	477	61.7	154	21.2	130	17.1	
2	545	12.1	361	66.6	118	21.7	66	11.7	
≥3	161	4.4	87	53.5	45	29.6	29	17.0	
**Children’s age (years)^d^**									
<1	96	7.9	63	59.4	18	24.7	15	15.9	0.866
1−5	522	38.6	335	63.7	110	21.6	77	14.7	0.837
6−12	786	53.2	483	61.4	191	25.2	112	13.4	0.033
13−17	559	37.4	357	64.0	115	21.0	87	15.0	0.711
**Cigarettes smoked per day**									<0.001
≤ 10	1417	33.2	1040	72.2	224	16.3	153	11.5	
11−20	1953	52.8	1130	56.2	477	26.6	346	17.2	
21−30	321	8.9	167	55.4	76	22.5	78	22.1	
>30	189	5.1	70	39.5	61	30.7	58	29.8	
**Nicotine dependence**									<0.001
Low	1619	40.0	1127	68.2	297	19.3	195	12.5	
Medium	1727	51.1	967	54.9	419	26.1	341	19.0	
High	312	9.0	132	45.7	93	28.3	87	26.0	
**Attempts to quit smoking**									<0.001
Yes	2150	53.4	1409	64.5	423	20.9	318	14.6	
No	1734	46.6	999	56.6	416	25.1	319	18.3	

a Weighted percentages per column.

b Weighted percentages per row.

c χ^2^ test.

d Multiple response.

Further analysis comparing smoking rules in cars (partial vs total ban, and no rules vs total ban) adjusting for potential confounders showed that, compared to Germany, only smokers from Greece were more likely to have a partial smoking ban rather than a total ban (PR=1.67; 95% CI: 1.20-2.34; Table 4). Smokers from all countries, except Hungary, had a higher prevalence of not having smoking rules than having a total smoking ban in cars, compared to Germany. As shown in Table 4, having no-smoking rules in cars was more likely among smokers at younger ages, low educational level, who smoked more CPD, with medium nicotine dependence, and with no attempts to quit smoking ever.

**Table 4:** Comparison of smoking rules (partial ban vs total ban; no rules vs total ban) in smokers’ cars in presence of children according to independent variables, 2016

	*Partial ban vs Total ban*			*No rules vs Total ban*		
	*PR*	*95% CI*	*p^a^*	*PR*	*95% CI*	*p^a^*
**Country**						
Germany	1	-		1	-	
Greece	1.67	1.20−2.34	0.003	1.68	1.10−2.59	0.018
Hungary	0.98	0.67−1.43	0.921	1.48	0.94−2.35	0.093
Poland	0.98	0.67−1.44	0.912	1.80	1.12−2.88	0.015
Romania	1.44	0.98−2.11	0.061	1.86	1.18−2.94	0.007
Spain	1.00	0.65−1.54	0.993	2.06	1.34−3.16	0.001
**Sex**						
Men	1	-		1	-	
Women	0.89	0.77−1.03	0.110	0.87	0.73−1.04	0.125
**Age (years)**						
18−24	1.65	1.07−2.55	0.025	1.64	1.04−2.58	0.033
25−39	1.29	1.01−1.65	0.039	1.36	1.04−1.77	0.024
40−54	1.45	1.19−1.76	<0.001	1.21	0.96−1.52	0.099
≥55	1	-		1	-	
**Educational level**						
Low	1.20	0.92−1.57	0.176	1.55	1.09−2.20	0.014
Intermediate	1.11	0.88−1.41	0.381	1.22	0.87−1.72	0.249
High	1	-		1	-	
**Smoker partner**						
Yes	1.04	0.89−1.23	0.606	1.10	0.88−1.37	0.411
No	1	-		1	-	
**Children**						
Yes	1	-		1	-	
No	1.13	0.94−1.34	0.194	1.22	0.99−1.50	0.061
**Cigarettes smoked per day**						
≤10	1	-		1	-	
11−20	1.74	1.33−2.27	<0.001	1.46	1.06−1.99	0.019
21−30	1.30	0.88−1.90	0.184	1.81	1.19−2.75	0.005
>30	1.45	0.85−2.46	0.169	2.66	1.40−5.05	0.003
**Nicotine dependence**						
Low	1	-		1	-	
Medium	1.01	0.79−1.29	0.917	1.40	1.06−1.85	0.018
High	1.47	0.92−2.33	0.105	1.25	0.72−2.16	0.423
**Attempts to quit smoking**						
Yes	1	-		1	-	
No	1.12	0.96−1.31	0.141	1.28	1.06−1.54	0.009

Prevalence ratios (PR) are derived from Poisson regression models, adjusted for all independent variables.

a χ^2^ test.

## DISCUSSION

More than a quarter of smokers surveyed across six EU MS reported having a total smoking ban in their homes; a similar proportion of smokers had no smoking rules. Prevalence of total smoking ban in homes varied across countries, from 13.1% in Spain to 35.5% in Hungary. These findings are consistent with data from other studies conducted in European countries within the ITC Project^17-20^ that indicated cross-country differences in the prevalence of smoke-free homes, varying from 13% in Scotland in 2007 to 38.2% in Germany in 2009. The later studies also indicated an increased prevalence of smoke-free homes in European countries over two survey waves^17-20^.

A different situation was found in cars when children were present where 61% of smokers had a total smoking ban and only 16% had no rules, with less heterogeneity observed across countries, varying from 51.8% in Greece to 67.7% in Germany. Data from other ITC studies conducted between 2007 and 2008 in France, Germany and the Netherlands indicated generally lower support for smoking bans in cars when children are present (41%, 48%, 64%, respectively)^21^, probably due to the time that has elapsed between these ITC studies and ours. In a brief review that included eight studies examining attitudes of adult smokers to smoking restrictions in cars with children^22^, the authors found that support for such regulations generally increased with time, for example, from 63%, 45% and 51% of surveyed smokers in New South Wales (Australia) in 1994, 2000 and 2004, respectively, to 96% in New Zealand in 2007-2008 and 87% in South Australia (Australia) in 2008^22^.

It is noteworthy to mention some differences observed in the prevalence of partial smoking rules and no-smoking rules in homes. For example, we observed that the higher the educational level, the higher the prevalence of partial bans in homes and the lower the prevalence of no rules. Also, the trend in the prevalence of the different rules differed by sex, age group, educational level and having children. The prevalence of no-smoking rules among participants who had children was almost half the prevalence of no-smoking rules among those who did not have children; conversely, the prevalence of partial bans was slightly higher among those who had children. In cars, such differences in prevalence are not remarkable, but we still observed some by educational level in a similar way to what we observed in homes. By CPD, the more the CPD smoked the more the prevalence of partial smoking rules and no-smoking rules; but the increase is two-fold for partial rules, whereas it is three-fold for no rules.

In our study, some of the factors that seem to affect smokers’ support for smoke-free regulations in these private settings are: having higher educational level, having children, and having a non-smoker partner. Age seems to have a different effect on the support for smoking regulation in these settings; higher support for smoke-free homes was observed in younger people and higher support for smoke-free cars in older people. Our data also showed that a total smoking ban in homes and cars was more likely among smokers with higher educational level, as shown by others^23,24^. This possibly indicates that the adoption of voluntary smoke-free rules among smokers follows diffusion of innovations theory (early adopters are those from more advantaged socioeconomic groups)^25^. Although having children is taken into account in deciding to have both partial and total smoking bans in both settings, we observed that this factor was more important for total smoking bans. This finding has potential implications for the encouragement of smokers with partial smoking bans to move towards total smoking bans in their homes and cars. In addition, having a non-smoker partner was also associated with smoking bans in homes and cars, indicating the importance of social support in encouraging smoke-free environments in private settings. The social system is crucial for the diffusion of smoking bans, because decisions are made not only at the individual but also at the collective and societal level^25^, thus highlighting the importance of the interaction between the individual and the environment^26^.

The results show, however, that some improvements are needed to protect non-smokers from second-hand smoke exposure in private settings, especially vulnerable members of the population, such as children. It has been proven that partial smoking does not effectively protect non-smokers from second-hand tobacco smoke due to leaking smoke^27-29^; furthermore, smoking in this context is still being promoted as a social norm. Furthermore, our findings demonstrate differences among smokers across the six EU MS in the attempt to implement smoking bans in private settings. Cross-country variation might reflect the level of denormalization of smoking in each country. Furthermore, differences may be related to the implementation status of smoke-free legislation, namely Article 8 of the FCTC, within each EU MS. While smoke-free legislation generally does not have wide jurisdiction over private settings, several studies have demonstrated the positive impact of smoke-free legislation on these unregulated places^10,11,30-33^. Comprehensive population-level policies may increase the prevalence of smoke-free environments in private settings^33,34^ by promoting awareness of the harmful effects of second-hand smoke exposure and the beneficial effects of reducing such exposure. Among the EU MS participating in our study, Germany and Poland are the countries were the least proportion of the population is protected by smoke-free laws regulating public places (national regulation is absent in Germany, while Poland has few public places where smoking is prohibited by national law)^35^. When examining the prevalence of total or partial smoking bans by country, no clear pattern in relation to smoke-free environment legislation was observed; therefore, further longitudinal analyses are necessary to better explain the effects of smoke-free regulations and voluntary smoking bans in private settings. Also, studies should take into account the degree to which public regulations are enforced (overall and by country) and its relationship to other issues, such as social conditions that may affect smoking bans in private settings.

Disentangling the relationship and causal pathway between the awareness of second-hand smoke risks, the country-level climate of smoking denormalization, the degree of implementation of smoke-free legislation, and the individual opting for smoke-free homes and cars, are challenging issues that warrant further research. A comprehensive understanding of the factors associated with the voluntary implementation of smoke-free rules in private settings and the mechanism of impact on the population, can contribute to enhancing and tailoring interventions.

This study has some limitations. First, this is a survey conducted among smokers; thus, some information bias on smoking rules is possible due to social desirability and the general antismoking climate, with country variations across EU-MS. These results, therefore, cannot be generalised to all populations, such as non-smokers. Voluntary smoke-free rules in homes and cars are, however, more frequent among non-smokers^36-40^. Second, survey questions on smoking rules in cars were asked specifically when children were present in the car, indicating that smoking may still occur in the absence of minors in the car. Third, the cross-sectional nature of the analysis precludes any inference about cause-effect relationships between the variables studied. Fourth, the use of self-reported information may introduce a source of bias that can affect the validity of our results. Nevertheless, self-reporting of smoking bans has been shown to have moderate to high correlation with measures of airborne nicotine, with excellent specificity and positive predictive values^29^. Also, the use of questionnaires is extensive in population studies, because it allows reaching many people at low cost.

On the other hand, the study has many strengths, including a large sample size, the representativeness of the samples of smokers in each country, and the use of a common questionnaire, derived from the expansive ITC Project^14^. Moreover, the use of six EU MS with diverse geographical and sociodemographic characteristics provided variability in the implementation extent of smoke-free rules in private settings. These EU MS have different smoking prevalence and different stages of the tobacco epidemic (all of them at advanced stages, III or IV of the tobacco epidemic model)^41^. In addition, these countries share some legislation, such as the EU Tobacco Products Directive, whilst maintaining their own legislation. This study provides a baseline assessment for analysis of prospective data in successive waves of the EUREST-PLUS cohort study that will allow trends to be examined. The current analysis will further facilitate evaluation of changes in voluntary smoke-free rules in private settings, and other smoking-related variables, as a result of changes in legislation.

## CONCLUSIONS

This study found that a quarter of smokers have a voluntary smoking ban at home and almost two-thirds for cars in the presence of children. These figures, however, are far from satisfactory and highlight the need to promote public policies and interventions aimed at increasing the number of smoke-free homes and cars.
