# Supplementary material for: Prevalence and correlates of different smoking bans in homes and cars among smokers in six countries of the EUREST-PLUS ITC Europe Surveys
Source: Tob Induc Dis. 2019 Jan 31;16:A8. doi: 10.18332/tid/94827 (PMC6661853; doi:10.18332/tid/94827)
Supplement: Supplementary file 1 [file TID-16-A8-s1.pdf]

Supplementary Table 1. Prevalence of smoking rules in smokers' homes and cars with children, 2016

	**Smoking rules in homes**						**Smoking rules in cars with children**					
	Total ban		Partial ban		No rules		Total ban		Partial ban		No rules	
	n	%	n	%	n	%	n	%	n	%	n	%
**Overall**	1686	26.5	2661	44.7	1620	28.8	2409	60.9	839	22.9	637	16.2
Germany	322	30.6	451	45.7	230	23.7	498	67.7	142	21.1	75	11.2
Greece	197	17.2	493	49.9	310	32.9	389	51.8	245	32.2	133	16.0
Hungary	365	35.5	418	42.2	211	22.3	350	63.3	97	18.1	99	18.6
Poland	300	30.0	402	42.5	270	27.5	433	64.9	103	19.2	99	15.9
Romania	355	33.1	463	46.7	180	20.2	303	57.5	138	26.6	88	15.9
Spain	147	13.1	434	41.3	419	45.6	436	61.5	114	18.1	143	20.4
p[a]			<0.001						<0.001			

All are weighted percentages per row.
[a] χ^2 test.

© 2019 Fu M.
