# Supplementary material for: Prevalence and correlates of different smoking bans in homes and cars among smokers in six countries of the EUREST-PLUS ITC Europe Surveys
Source: Tob Induc Dis. 2019 Jan 31;16:A8. doi: 10.18332/tid/94827 (PMC6661853; doi:10.18332/tid/94827)
Supplement: Supplementary file 2 [file TID-16-A8-s2.pdf]

Supplementary Table 2. Prevalence of smoking rules in smokers' homes by country, according to sociodemographic and smoking characteristics, 2016

Country: Germany

	Overall		Smoking rules in homes						p[c]
			Total ban		Partial rules		No rules		
	n	%[a]	n	%[b]	n	%[b]	n	%[b]	
**Overall**	1003	100	322	30.6	451	45.7	230	23.7	<0.001
**Sex**									0.005
Men	507	60.9	154	29.2	215	43.3	138	27.5	
Women	496	39.1	168	32.6	236	49.5	92	17.9	
**Age (years)**									<0.001
18−24	88	8.4	37	39.2	31	34.5	20	26.3	
25−39	283	25.6	110	36.4	124	47.5	49	16.1	
40−54	339	36.5	112	32.2	153	43.4	74	24.4	
≥ 55	293	29.5	63	21.0	143	50.2	87	28.8	
**Educational level**									0.013
Low	509	49.6	146	28.9	227	43.4	136	27.7	
Intermediate	417	42.5	153	34.0	182	45.7	82	20.3	
High	75	7.9	23	23.4	40	58.9	12	17.7	
**Smoker partner**									
Yes	385	63.6	132	32.3	182	47.1	71	20.6	0.005
No	212	36.4	97	45.3	93	46.0	22	8.7	
**Children**									<0.001
Yes	300	28.8	142	44.8	138	47.5	20	7.7	
No	701	71.2	179	24.8	312	45.0	210	30.2	
**Number of children**									<0.001
0	701	71.2	179	24.8	312	45.0	210	30.2	
1	158	14.4	67	41.1	76	47.0	15	11.9	
2	111	11.0	60	49.9	47	46.2	4	3.9	
≥3	31	3.4	15	43.7	15	54.0	1	2.3	
**Children's age (years)[d]**									
<1	14	6.3	9	-	5	-	0	-	0.331
1−5	100	34.1	55	53.0	38	40.9	7	6.1	0.137
6−12	147	49.9	76	47.9	66	47.3	5	4.8	0.054
13−17	132	44.4	55	40.3	67	51.1	10	8.6	0.217
**Cigarettes smoked per day**									<0.001
≤10	376	36.6	157	40.5	164	43.9	55	15.6	
11−20	479	49.2	138	26.7	215	46.7	126	26.6	
21−30	110	10.5	22	19.6	49	45.2	39	35.2	
>30	36	3.7	3	11.9	23	54.5	10	33.6	
**Nicotine dependence**									<0.001
Low	440	50.1	163	35.5	192	45.5	85	19.0	
Medium	381	43.1	98	23.7	165	44.2	118	32.1	
High	61	6.8	6	12.1	37	56.2	18	31.7	
**Attempts to quit smoking**									<0.001
Yes	555	54.5	208	36.4	233	42.8	114	20.8	
No	448	45.5	114	23.5	218	49.2	116	27.3	

[a] Weighted percentages per column.
[b] Weighted percentages per row.
[c] χ^2 test.
[d] Multiple response.

Country: Greece

	Overall		Smoking rules in homes						p^c
			Total ban		Partial rules		No rules		
	n	%[a]	n	%[b]	n	%[b]	n	%[b]	
**Overall**	1000	100	197	17.2	493	49.9	310	32.9	<0.001
**Sex**									0.016
Men	544	53.2	121	19.3	247	45.2	176	35.5	
Women	456	46.8	76	14.9	246	55.1	134	30.0	
**Age (years)**									0.003
18−24	61	8.4	10	13.7	18	28.4	33	57.9	
25−39	255	28.8	54	17.2	118	46.4	83	36.4	
40−54	383	35.6	75	18.6	204	53.6	104	27.8	
≥ 55	301	27.2	58	16.6	153	55.2	90	28.2	
**Educational level**									0.001
Low	306	30.2	47	12.5	143	49.2	116	38.3	
Intermediate	488	49.0	102	18.9	235	47.4	151	33.7	
High	203	20.8	47	20.1	114	56.9	42	23.0	
**Smoker partner**									0.005
Yes	409	58.9	74	17.3	225	56.1	110	26.6	
No	275	41.1	79	24.8	134	52.5	62	22.7	
**Children**									<0.001
Yes	306	29.1	98	31.6	151	48.4	57	20.0	
No	692	70.9	99	11.4	341	50.5	252	38.1	
**Number of children**									<0.001
0	692	70.9	99	11.4	341	50.5	252	38.1	
1	132	13.0	40	29.7	69	48.8	23	21.5	
2	133	12.4	49	35.2	62	48.8	22	16.0	
≥3	41	3.7	9	26.0	20	45.6	12	28.4	
**Children's age (years)[d]**									
<1	20	7.5	10	47.3	8	45.8	2	6.9	0.183
1−5	113	39.4	49	40.0	42	37.3	22	22.7	0.002
6−12	174	56.8	55	30.6	84	49.4	35	20.0	0.743
13−17	109	33.1	26	26.8	60	52.3	23	20.9	0.074
**Cigarettes smoked per day**									0.005
≤10	276	28.6	70	21.1	124	47.2	82	31.7	
11−20	456	46.4	79	15.9	246	52.5	131	31.6	
21−30	131	12.4	28	18.9	64	51.6	39	29.5	
>30	137	12.6	20	11.7	59	44.7	58	43.6	
**Nicotine dependence**									<0.001
Low	322	34.0	82	22.3	149	48.6	91	29.1	
Medium	475	49.8	86	16.0	254	52.8	135	31.2	
High	173	16.2	24	11.3	76	45.4	73	43.3	
**Attempts to quit smoking**									0.123
Yes	429	43.8	95	19.7	213	51.8	121	28.5	
No	571	56.2	102	15.3	280	48.4	189	36.3	

[a] Weighted percentages per column.
[b] Weighted percentages per row.
[c] χ^2 test.
[d] Multiple response.

Country: Hungary

	Overall		Smoking rules in homes						
			Total ban		Partial rules		No rules		p[c]
	n	%[a]	n	%[b]	n	%[b]	n	%[b]	
**Overall**	994	100	365	35.5	418	42.2	211	22.3	<0.001
**Sex**									0.348
Men	517	59.1	187	34.0	211	42.1	119	23.9	
Women	477	40.9	178	37.5	207	42.4	92	20.1	
**Age (years)**									0.003
18−24	59	9.3	24	36.4	24	43.4	11	20.2	
25−39	280	33.9	119	42.2	120	41.3	41	16.5	
40−54	354	33.5	127	32.3	155	44.3	72	23.4	
≥ 55	301	23.3	95	29.9	119	40.1	87	30.0	
**Educational level**									0.054
Low	614	64.7	208	32.3	266	42.8	140	24.9	
Intermediate	308	29.2	122	40.1	127	41.7	59	18.2	
High	70	6.1	35	48.6	25	39.0	10	12.4	
**Smoker partner**									0.009
Yes	375	57.2	123	30.6	167	45.3	85	24.1	
No	284	42.8	125	43.4	112	39.6	47	17.0	
**Children**									0.002
Yes	325	35.3	139	41.4	136	42.5	50	16.1	
No	669	64.7	226	32.2	282	42.0	161	25.8	
**Number of children**									<0.001
0	669	64.7	226	32.2	282	42.0	161	25.8	
1	139	14.2	68	46.9	58	44.9	13	8.2	
2	118	12.5	53	43.8	42	33.4	23	22.8	
≥3	68	8.6	18	28.9	36	51.6	14	19.5	
**Children's age (years)[d]**									
<1	31	11.8	11	47.3	15	41.0	5	11.7	0.674
1−5	141	45.8	58	39.9	57	40.6	26	19.5	0.409
6−12	180	56.4	77	40.3	77	43.3	26	16.4	0.854
13−17	132	36.6	54	39.1	57	45.2	21	15.7	0.855
**Cigarettes smoked per day**									<0.001
≤10	316	30.0	153	48.5	118	37.0	45	14.5	
11−20	575	58.7	192	32.1	254	44.2	129	23.7	
21−30	76	8.5	15	17.3	32	43.7	29	39.0	
>30	27	2.8	5	21.9	14	50.0	8	28.1	
**Nicotine dependence**									<0.001
Low	347	35.4	156	45.9	137	38.8	54	15.3	
Medium	567	57.1	187	30.1	248	44.3	132	25.6	
High	68	7.5	15	22.5	29	44.7	24	32.8	
**Attempts to quit smoking**									0.786
Yes	453	44.5	170	36.2	191	41.2	92	22.6	
No	541	55.5	195	34.8	227	43.0	119	22.2	

[a] Weighted percentages per column.
[b] Weighted percentages per row.
[c] χ^2 test.
[d] Multiple response.

Country: Poland

	Overall		Smoking rules in homes: Total ban		Partial rules		No rules		p[c]
	n	%[a]	n	%[b]	n	%[b]	n	%[b]	
**Overall**	972	100	300	30.0	402	42.5	270	27.5	<0.001
**Sex**									0.599
Men	463	55.5	143	29.5	185	41.1	135	29.5	
Women	509	44.5	157	30.6	217	44.2	135	25.2	
**Age (years)**									<0.001
18−24	72	8.0	23	30.9	33	46.1	16	23.0	
25−39	330	33.5	119	34.6	144	45.8	67	19.6	
40−54	272	29.5	79	29.7	115	41.4	78	28.9	
≥ 55	298	29.0	79	24.6	110	38.7	109	36.7	
**Educational level**									<0.001
Low	123	11.8	25	19.1	44	37.7	54	43.2	
Intermediate	728	77.5	212	28.5	321	45.2	195	26.3	
High	107	10.7	59	55.1	33	29.6	15	15.3	
**Smoker partner**									<0.001
Yes	371	58.5	116	33.4	147	38.8	108	27.8	
No	269	41.5	100	34.3	127	49.3	42	16.4	
**Children**									<0.001
Yes	336	34.3	129	39.6	151	46.2	56	14.2	
No	633	65.7	171	25.2	250	40.6	212	34.2	
**Number of children**									<0.001
0	633	65.7	171	25.2	250	40.6	212	34.2	
1	181	18.9	69	40.9	82	45.7	30	13.4	
2	119	11.4	51	44.2	50	43.9	18	11.9	
≥3	36	4.0	9	19.6	19	55.1	8	25.3	
**Children's age group[d]**									
<1	27	8.3	10	30.9	13	58.4	4	10.7	0.932
1−5	117	33.5	47	41.3	48	42.0	22	16.7	0.535
6−12	172	54.0	61	34.8	82	51.1	29	14.1	0.501
13−17	125	35.8	45	36.2	57	45.4	23	18.4	0.714
**Cigarettes smoked per day**									<0.001
≤10	323	31.2	135	42.4	136	42.3	52	15.3	
11−20	550	58.6	151	26.1	231	44.1	168	29.8	
21−30	67	7.8	10	20.1	24	34.5	33	45.4	
>30	26	2.4	3	5.9	9	33.8	14	60.3	
**Nicotine dependence**									<0.001
Low	350	36.6	149	42.9	136	38.9	65	18.2	
Medium	501	57.6	122	24.4	218	44.3	161	31.3	
High	57	5.8	5	5.1	21	36.6	31	58.3	
**Attempts to quit smoking**									0.055
Yes	544	53.5	181	32.3	227	44.5	136	23.2	
No	425	46.5	118	27.2	174	40.1	133	32.7	

[a] Weighted percentages per column.

[b] Weighted percentages per row.

[c] χ^2 test.

[d] Multiple response.

Country: Romania

	Overall		Total ban		Partial rules		No rules		p[c]
			**Smoking rules in homes**						
	n	%[a]	n	%[b]	n	%[b]	n	%[b]	
**Overall**	998	100	355	33.1	463	46.7	180	20.2	<0.001
**Sex**									0.006
Men	580	58.4	228	35.3	246	43.9	106	20.8	
Women	418	41.6	127	29.8	217	50.7	74	19.5	
**Age (years)**									0.041
18−24	110	14.3	46	38.1	43	38.2	21	23.7	
25−39	298	38.2	101	29.7	135	46.9	62	23.4	
40−54	321	30.9	98	32.7	170	51.6	53	15.7	
≥ 55	269	16.6	110	36.9	115	44.5	44	18.6	
**Educational level**									0.001
Low	245	24.8	86	30.7	98	41.1	61	28.2	
Intermediate	627	63.0	234	34.4	288	46.5	105	19.1	
High	114	12.2	32	32.1	68	55.8	14	12.1	
**Smoker partner**									<0.001
Yes	357	57.7	92	24.0	194	52.7	71	23.3	
No	343	42.3	152	43.8	151	43.8	40	12.4	
**Children**									0.068
Yes	356	38.9	135	35.1	170	49.1	51	15.8	
No	641	61.1	219	31.6	293	45.3	129	23.1	
**Number of children**									0.045
0	641	61.1	219	31.6	293	45.3	129	23.1	
1	180	19.7	76	37.4	84	51.7	20	10.9	
2	131	15.0	45	33.2	67	49.1	19	17.7	
≥3	45	4.2	14	30.7	19	37.0	12	32.3	
**Children's age (years)[d]**									
<1	28	7.6	16	56.6	10	34.1	2	9.3	0.083
1−5	143	43.8	53	34.4	72	51.1	18	14.5	0.641
6−12	190	54.3	60	29.9	99	50.3	31	19.8	0.029
13−17	143	36.5	52	35.0	65	46.8	26	18.2	0.235
**Cigarettes smoked per day**									<0.001
≤10	355	32.5	155	43.6	162	45.3	38	11.1	
11−20	528	55.9	166	29.0	258	47.5	104	23.5	
21−30	69	6.9	18	18.3	28	50.5	23	31.2	
>30	46	4.7	16	29.6	15	42.5	15	27.9	
**Nicotine dependence**									<0.001
Low	371	34.5	156	42.9	170	44.2	45	12.9	
Medium	494	56.6	152	27.9	239	48.0	103	24.1	
High	80	8.9	21	22.8	30	45.5	29	31.7	
**Attempts to quit smoking**									0.021
Yes	620	62.4	235	35.7	288	46.8	97	17.5	
No	378	37.6	120	28.5	175	46.7	83	24.8	

[a] Weighted percentages per column.
[b] Weighted percentages per row.
[c] χ^2 test.
[d] Multiple response.

Country: Spain

	Overall		Smoking rules in homes						p^c
			Total ban		Partial rules		No rules		
	n	%[a]	n	%[b]	n	%[b]	n	%[b]	
**Overall**	1000	100	147	13.1	434	41.3	419	45.6	<0.001
**Sex**									0.151
Men	544	57.3	77	11.9	224	40.0	243	48.1	
Women	456	42.7	70	14.8	210	43.0	176	42.1	
**Age (years)**									0.011
18−24	117	12.1	21	13.7	39	31.8	57	54.5	
25−39	311	29.0	50	15.4	141	44.2	120	40.4	
40−54	323	38.5	43	11.6	160	44.5	120	43.9	
≥ 55	249	20.4	33	12.5	94	36.9	122	50.6	
**Educational level**									0.047
Low	410	44.2	51	9.6	173	39.1	186	51.3	
Intermediate	506	47.9	86	16.8	229	43.6	191	39.6	
High	83	7.9	10	11.1	31	39.0	42	49.9	
**Smoker partner**									<0.001
Yes	345	57.6	32	8.3	173	47.0	140	44.7	
No	246	42.4	61	23.4	120	44.0	65	32.6	
**Children**									<0.001
Yes	335	35.1	59	16.9	183	49.4	93	33.7	
No	665	64.9	88	11.1	251	36.9	326	52.0	
**Number of children**									<0.001
0	665	64.9	88	11.1	251	36.9	326	52.0	
1	210	22.1	32	16.0	124	55.8	54	28.2	
2	97	10.3	22	20.1	46	37.7	29	42.2	
≥3	28	2.7	5	11.9	13	42.6	10	45.5	
**Children's age group**[d]									
<1	20	5.9	3	11.4	14	65.4	3	23.2	0.325
1−5	117	33.7	25	19.3	62	50.0	30	30.7	0.403
6−12	166	47.6	31	14.8	97	52.6	38	32.6	0.143
13−17	119	38.4	23	19.9	50	37.3	46	42.8	0.001
**Cigarettes smoked per day**									<0.001
≤10	417	40.1	94	21.9	188	43.4	135	34.7	
11−20	471	48.1	45	7.7	201	39.6	225	52.7	
21−30	70	7.2	4	4.3	27	42.5	39	53.2	
>30	42	4.6	4	7.6	18	38.5	20	53.9	
**Nicotine dependence**									<0.001
Low	511	50.0	96	17.6	237	45.3	178	37.1	
Medium	381	41.6	35	7.5	153	37.4	193	55.1	
High	77	8.4	3	3.4	29	35.5	45	61.1	
**Attempts to quit smoking**									0.002
Yes	622	61.7	101	14.5	287	44.8	234	40.7	
No	378	38.3	46	11.0	147	35.7	185	53.3	

[a] Weighted percentages per column.
[b] Weighted percentages per row.
[c] χ^2 test.
[d] Multiple response.

© 2019 Fu M.
