# Supplementary material for: Prevalence and correlates of different smoking bans in homes and cars among smokers in six countries of the EUREST-PLUS ITC Europe Surveys
Source: Tob Induc Dis. 2019 Jan 31;16:A8. doi: 10.18332/tid/94827 (PMC6661853; doi:10.18332/tid/94827)
Supplement: Supplementary file 3 [file TID-16-A8-s3.pdf]

***Supplementary Table 3. Prevalence of smoking rules in smokers' cars with children by country, according to sociodemographic and smoking characteristics, 2016***

**Country: Germany**

| | Overall | | Smoking rules in cars with children | | | | | | $p^c$ |
|---|---|---|---|---|---|---|---|---|---|
| | Overall | | Total ban | | Partial rules | | No rules | | |
| | n | %[a] | n | %[b] | n | %[b] | n | %[b] | |
| **Overall** | 715 | 16.7 | 498 | 67.7 | 142 | 21.1 | 75 | 11.2 | <0.001 |
| **Sex** | | | | | | | | | 0.032 |
| Men | 356 | 60.9 | 233 | 64.4 | 77 | 22.9 | 46 | 12.7 | |
| Women | 359 | 39.1 | 265 | 72.9 | 65 | 18.1 | 29 | 9.0 | |
| **Age (years)** | | | | | | | | | 0.593 |
| 18−24 | 43 | 8.4 | 27 | 60.2 | 10 | 23.1 | 6 | 16.7 | |
| 25−39 | 209 | 25.6 | 143 | 68.3 | 47 | 22.4 | 19 | 9.3 | |
| 40−54 | 264 | 36.5 | 181 | 66.7 | 51 | 20.3 | 32 | 13.0 | |
| ≥ 55 | 199 | 29.5 | 147 | 70.0 | 34 | 20.5 | 18 | 9.5 | |
| **Educational level** | | | | | | | | | 0.919 |
| Low | 348 | 49.6 | 244 | 69.2 | 67 | 19.8 | 37 | 11.0 | |
| Intermediate | 309 | 42.4 | 212 | 66.3 | 63 | 21.5 | 34 | 12.2 | |
| High | 56 | 8.0 | 40 | 65.6 | 12 | 26.7 | 4 | 7.7 | |
| **Smoker partner** | | | | | | | | | 0.100 |
| Yes | 309 | 63.6 | 219 | 70.1 | 60 | 20.5 | 30 | 9.4 | |
| No | 178 | 36.4 | 141 | 73.3 | 22 | 17.4 | 15 | 9.3 | |
| **Children** | | | | | | | | | 0.012 |
| Yes | 262 | 28.8 | 189 | 71.6 | 57 | 22.1 | 16 | 6.3 | |
| No | 451 | 71.2 | 308 | 65.7 | 84 | 20.3 | 59 | 14.0 | |
| **Number of children** | | | | | | | | | 0.073 |
| 0 | 451 | 71.2 | 308 | 65.7 | 84 | 20.3 | 59 | 14.0 | |
| 1 | 131 | 14.4 | 90 | 69.6 | 33 | 25.2 | 8 | 5.2 | |
| 2 | 101 | 11.0 | 78 | 76.3 | 17 | 16.4 | 6 | 7.3 | |
| ≥3 | 30 | 3.4 | 21 | 64.7 | 7 | 28.4 | 2 | 6.9 | |
| **Children's age (years)[d]** | | | | | | | | | |
| <1 | 13 | 6.3 | 8 | 70.2 | 4 | 25.8 | 1 | 4.0 | 0.677 |
| 1−5 | 85 | 34.1 | 65 | 76.0 | 17 | 21.2 | 3 | 2.8 | 0.392 |
| 6−12 | 134 | 49.9 | 96 | 70.1 | 30 | 24.4 | 8 | 5.5 | 0.967 |
| 13−17 | 120 | 44.4 | 90 | 72.2 | 23 | 20.4 | 7 | 7.4 | 0.619 |
| **Cigarettes smoked per day** | | | | | | | | | <0.001 |
| ≤10 | 280 | 36.6 | 226 | 79.1 | 38 | 14.4 | 16 | 6.5 | |
| 11−20 | 341 | 49.2 | 222 | 63.1 | 83 | 25.6 | 36 | 11.3 | |
| 21−30 | 74 | 10.5 | 42 | 55.5 | 17 | 24.7 | 15 | 19.8 | |
| >30 | 18 | 3.7 | 7 | 34.1 | 3 | 16.1 | 8 | 49.8 | |
| **Nicotine dependence** | | | | | | | | | <0.001 |
| Low | 323 | 50.1 | 239 | 71.1 | 59 | 20.7 | 25 | 8.2 | |
| Medium | 259 | 43.1 | 160 | 60.6 | 62 | 23.8 | 37 | 15.6 | |
| High | 36 | 6.8 | 17 | 44.7 | 8 | 21.2 | 11 | 34.1 | |
| **Attempts to quit smoking** | | | | | | | | | 0.008 |
| Yes | 424 | 54.5 | 314 | 74.1 | 72 | 16.5 | 38 | 9.4 | |
| No | 291 | 45.5 | 184 | 58.7 | 70 | 27.5 | 37 | 13.8 | |

[a] Weighted percentages per column.
[b] Weighted percentages per row.
[c] $\chi^2$ test.
[d] Multiple response.

**Country: Greece**

| | Overall | | Smoking rules in cars with children | | | | | | p[c] |
|---|---|---|---|---|---|---|---|---|---|
| | | | Total ban | | Partial rules | | No rules | | |
| | n | %[a] | n | %[b] | n | %[b] | n | %[b] | |
| **Overall** | 767 | 16.6 | 389 | 51.8 | 245 | 32.2 | 133 | 16.0 | <0.001 |
| **Sex** | | | | | | | | | 0.009 |
| Men | 430 | 53.2 | 201 | 46.1 | 140 | 34.2 | 89 | 19.7 | |
| Women | 337 | 46.8 | 188 | 58.7 | 105 | 29.9 | 44 | 11.4 | |
| **Age (years)** | | | | | | | | | 0.243 |
| 18−24 | 41 | 8.4 | 21 | 52.6 | 12 | 24.4 | 8 | 23.0 | |
| 25−39 | 195 | 28.9 | 97 | 53.1 | 64 | 31.7 | 34 | 15.2 | |
| 40−54 | 325 | 35.5 | 162 | 48.5 | 116 | 38.1 | 47 | 13.4 | |
| ≥ 55 | 206 | 27.2 | 109 | 55.2 | 53 | 26.1 | 44 | 18.7 | |
| **Educational level** | | | | | | | | | <0.001 |
| Low | 209 | 30.2 | 77 | 38.0 | 79 | 37.5 | 53 | 24.5 | |
| Intermediate | 391 | 49.0 | 216 | 55.8 | 113 | 30.0 | 62 | 14.2 | |
| High | 166 | 20.8 | 95 | 58.9 | 53 | 31.2 | 18 | 9.9 | |
| **Smoker partner** | | | | | | | | | 0.625 |
| Yes | 338 | 58.9 | 179 | 53.1 | 105 | 31.8 | 54 | 15.1 | |
| No | 237 | 41.1 | 119 | 52.6 | 73 | 32.1 | 45 | 15.3 | |
| **Children** | | | | | | | | | 0.466 |
| Yes | 285 | 29.1 | 142 | 49.4 | 98 | 35.0 | 45 | 15.6 | |
| No | 481 | 70.9 | 246 | 53.0 | 147 | 30.8 | 88 | 16.2 | |
| **Number of children** | | | | | | | | | 0.016 |
| 0 | 481 | 71.0 | 246 | 53.0 | 147 | 30.8 | 88 | 16.2 | |
| 1 | 124 | 13.0 | 66 | 51.0 | 38 | 32.7 | 20 | 16.3 | |
| 2 | 125 | 12.3 | 68 | 55.6 | 39 | 30.7 | 18 | 13.7 | |
| ≥3 | 36 | 3.7 | 8 | 21.1 | 21 | 58.9 | 7 | 20.0 | |
| **Children's age (years)[d]** | | | | | | | | | |
| <1 | 20 | 7.5 | 14 | 67.4 | 5 | 28.6 | 1 | 4.0 | 0.143 |
| 1−5 | 106 | 39.4 | 51 | 46.0 | 41 | 37.6 | 14 | 16.4 | 0.424 |
| 6−12 | 161 | 56.8 | 76 | 49.3 | 59 | 35.7 | 26 | 15.0 | 0.580 |
| 13−17 | 98 | 33.1 | 49 | 49.0 | 32 | 35.6 | 17 | 15.4 | 0.835 |
| **Cigarettes smoked per day** | | | | | | | | | <0.001 |
| ≤10 | 219 | 28.6 | 137 | 62.6 | 58 | 25.0 | 24 | 12.4 | |
| 11−20 | 345 | 46.4 | 171 | 49.7 | 113 | 34.7 | 61 | 15.6 | |
| 21−30 | 102 | 12.4 | 48 | 52.2 | 31 | 28.2 | 23 | 19.6 | |
| >30 | 101 | 12.6 | 33 | 32.6 | 43 | 45.0 | 25 | 22.4 | |
| **Nicotine dependence** | | | | | | | | | <0.001 |
| Low | 254 | 34.0 | 159 | 62.1 | 72 | 27.5 | 23 | 10.4 | |
| Medium | 362 | 49.8 | 169 | 47.3 | 114 | 33.2 | 79 | 19.5 | |
| High | 128 | 16.2 | 44 | 36.6 | 54 | 44.3 | 30 | 19.1 | |
| **Attempts to quit smoking** | | | | | | | | | 0.017 |
| Yes | 346 | 43.8 | 190 | 54.6 | 110 | 33.2 | 46 | 12.2 | |
| No | 421 | 56.2 | 199 | 49.4 | 135 | 31.5 | 87 | 19.1 | |

[a] Weighted percentages per column.
[b] Weighted percentages per row.
[c] $\chi^2$ test.
[d] Multiple response.

**Country: Hungary**

| | Overall | | Smoking rules in cars with children: Total ban | | Partial rules | | No rules | | p[c] |
|---|---|---|---|---|---|---|---|---|---|
| | n | %[a] | n | %[b] | n | %[b] | n | %[b] | |
| **Overall** | 546 | 16.6 | 350 | 63.3 | 97 | 18.1 | 99 | 18.6 | <0.001 |
| **Sex** | | | | | | | | | 0.145 |
| Men | 287 | 59.1 | 173 | 58.8 | 56 | 20.0 | 58 | 21.2 | |
| Women | 259 | 40.9 | 177 | 69.6 | 41 | 15.3 | 41 | 15.1 | |
| **Age (years)** | | | | | | | | | 0.699 |
| 18−24 | 28 | 9.2 | 16 | 45.5 | 5 | 26.4 | 7 | 28.1 | |
| 25−39 | 168 | 33.9 | 110 | 66.7 | 27 | 15.4 | 31 | 17.9 | |
| 40−54 | 208 | 33.6 | 129 | 60.0 | 44 | 21.2 | 35 | 18.8 | |
| ≥ 55 | 142 | 23.3 | 95 | 69.0 | 21 | 14.5 | 26 | 16.5 | |
| **Educational level** | | | | | | | | | 0.097 |
| Low | 309 | 64.7 | 192 | 59.6 | 55 | 19.6 | 62 | 20.8 | |
| Intermediate | 191 | 29.2 | 120 | 64.9 | 38 | 17.9 | 33 | 17.2 | |
| High | 45 | 6.1 | 37 | 84.4 | 4 | 7.1 | 4 | 8.5 | |
| **Smoker partner** | | | | | | | | | 0.082 |
| Yes | 218 | 57.2 | 130 | 58.8 | 41 | 19.2 | 47 | 22.0 | |
| No | 194 | 42.8 | 132 | 68.3 | 36 | 19.4 | 26 | 12.3 | |
| **Children** | | | | | | | | | |
| Yes | 203 | 35.3 | 131 | 62.2 | 40 | 21.2 | 32 | 16.6 | 0.429 |
| No | 343 | 64.7 | 219 | 64.1 | 57 | 15.8 | 67 | 20.1 | |
| **Number of children** | | | | | | | | | 0.813 |
| 0 | 343 | 64.6 | 219 | 64.1 | 57 | 15.8 | 67 | 20.1 | |
| 1 | 89 | 14.3 | 57 | 60.0 | 18 | 23.5 | 14 | 16.5 | |
| 2 | 74 | 12.5 | 48 | 66.2 | 16 | 18.8 | 10 | 15.0 | |
| ≥3 | 40 | 8.6 | 26 | 60.3 | 6 | 20.8 | 8 | 18.9 | |
| **Children's age (years)[d]** | | | | | | | | | |
| <1 | 16 | 11.8 | 9 | 47.5 | 4 | 33.0 | 3 | 19.5 | 0.767 |
| 1−5 | 74 | 45.8 | 49 | 65.2 | 10 | 16.6 | 15 | 18.2 | 0.148 |
| 6−12 | 122 | 56.4 | 77 | 63.0 | 27 | 22.2 | 18 | 14.8 | 0.547 |
| 13−17 | 92 | 36.6 | 61 | 66.7 | 17 | 17.5 | 14 | 15.8 | 0.886 |
| **Cigarettes smoked per day** | | | | | | | | | 0.070 |
| ≤10 | 184 | 30.0 | 132 | 69.7 | 27 | 14.8 | 25 | 15.5 | |
| 11−20 | 318 | 58.7 | 196 | 61.8 | 62 | 20.2 | 60 | 18.0 | |
| 21−30 | 34 | 8.5 | 17 | 51.0 | 6 | 16.5 | 11 | 32.5 | |
| >30 | 10 | 2.8 | 5 | 49.1 | 2 | 11.7 | 3 | 39.2 | |
| **Nicotine dependence** | | | | | | | | | 0.219 |
| Low | 206 | 35.4 | 140 | 67.0 | 35 | 17.5 | 31 | 15.5 | |
| Medium | 302 | 57.1 | 188 | 61.2 | 56 | 19.2 | 58 | 19.6 | |
| High | 32 | 7.5 | 17 | 55.9 | 5 | 12.2 | 10 | 31.9 | |
| **Attempts to quit smoking** | | | | | | | | | 0.216 |
| Yes | 247 | 44.5 | 168 | 68.5 | 40 | 17.1 | 39 | 14.4 | |
| No | 299 | 55.5 | 182 | 59.1 | 57 | 18.8 | 60 | 22.1 | |

[a] Weighted percentages per column.
[b] Weighted percentages per row.
[c] $\chi^2$ test.
[d] Multiple response.

**Country: Poland**

| | Overall | | Smoking rules in cars with children | | | | | | p[c] |
|---|---|---|---|---|---|---|---|---|---|
| | Overall | | Total ban | | Partial rules | | No rules | | |
| | n | %[a] | n | %[b] | n | %[b] | n | %[b] | |
| **Overall** | 635 | 16.7 | 433 | 64.9 | 103 | 19.2 | 99 | 15.9 | <0.001 |
| **Sex** | | | | | | | | | 0.472 |
| Men | 298 | 55.5 | 199 | 62.2 | 54 | 21.3 | 45 | 16.5 | |
| Women | 337 | 44.5 | 234 | 68.0 | 49 | 16.8 | 54 | 15.2 | |
| **Age (years)** | | | | | | | | | 0.200 |
| 18−24 | 42 | 8.0 | 25 | 59.4 | 11 | 24.5 | 6 | 16.1 | |
| 25−39 | 236 | 33.5 | 155 | 66.6 | 36 | 16.2 | 45 | 17.2 | |
| 40−54 | 182 | 29.5 | 124 | 61.4 | 33 | 23.7 | 25 | 14.9 | |
| ≥ 55 | 175 | 29.0 | 129 | 68.3 | 23 | 16.4 | 23 | 15.3 | |
| **Educational level** | | | | | | | | | 0.087 |
| Low | 53 | 11.8 | 37 | 71.5 | 6 | 12.5 | 10 | 16.0 | |
| Intermediate | 486 | 77.5 | 321 | 62.4 | 83 | 20.6 | 82 | 17.0 | |
| High | 89 | 10.7 | 71 | 76.8 | 11 | 12.5 | 7 | 10.7 | |
| **Smoker partner** | | | | | | | | | 0.401 |
| Yes | 268 | 58.5 | 192 | 70.0 | 37 | 16.6 | 39 | 13.4 | |
| No | 202 | 41.5 | 133 | 58.4 | 33 | 20.5 | 36 | 21.1 | |
| **Children** | | | | | | | | | 0.375 |
| Yes | 253 | 34.3 | 181 | 69.4 | 36 | 17.0 | 36 | 13.6 | |
| No | 380 | 65.7 | 252 | 62.2 | 66 | 20.3 | 62 | 17.5 | |
| **Number of children** | | | | | | | | | 0.637 |
| 0 | 380 | 65.7 | 252 | 62.2 | 66 | 20.3 | 62 | 17.5 | |
| 1 | 137 | 18.9 | 95 | 68.3 | 19 | 16.2 | 23 | 15.5 | |
| 2 | 94 | 11.4 | 68 | 69.9 | 15 | 19.0 | 11 | 11.1 | |
| ≥3 | 22 | 4.0 | 18 | 74.0 | 2 | 14.6 | 2 | 11.4 | |
| **Children's age (years)[d]** | | | | | | | | | |
| <1 | 19 | 8.3 | 14 | 69.3 | 3 | 25.2 | 2 | 5.5 | 0.884 |
| 1−5 | 87 | 33.5 | 66 | 76.9 | 10 | 11.1 | 11 | 12.0 | 0.528 |
| 6−12 | 133 | 54.0 | 90 | 60.9 | 19 | 20.4 | 24 | 18.7 | 0.177 |
| 13−17 | 90 | 35.8 | 64 | 71.2 | 15 | 18.6 | 11 | 10.2 | 0.612 |
| **Cigarettes smoked per day** | | | | | | | | | <0.001 |
| ≤10 | 240 | 31.2 | 190 | 77.5 | 25 | 11.3 | 25 | 11.2 | |
| 11−20 | 352 | 58.6 | 213 | 56.1 | 74 | 25.4 | 65 | 18.5 | |
| 21−30 | 33 | 7.8 | 26 | 82.2 | 2 | 7.5 | 5 | 10.3 | |
| >30 | 7 | 2.4 | 3 | 36.5 | 2 | 17.2 | 2 | 46.3 | |
| **Nicotine dependence** | | | | | | | | | <0.001 |
| Low | 254 | 36.6 | 201 | 77.5 | 23 | 9.0 | 30 | 13.5 | |
| Medium | 314 | 57.6 | 183 | 54.9 | 72 | 27.6 | 59 | 17.5 | |
| High | 21 | 5.8 | 16 | 81.4 | 2 | 4.5 | 3 | 14.1 | |
| **Attempts to quit smoking** | | | | | | | | | 0.354 |
| Yes | 362 | 53.5 | 255 | 67.2 | 55 | 19.1 | 52 | 13.7 | |
| No | 272 | 46.5 | 177 | 61.8 | 48 | 19.4 | 47 | 18.8 | |

[a] Weighted percentages per column.
[b] Weighted percentages per row.
[c] $\chi^2$ test.
[d] Multiple response.

**Country: Romania**

| | Overall | | Total ban | | Partial rules | | No rules | | $p^c$ |
|---|---|---|---|---|---|---|---|---|---|
| | | | **Smoking rules in cars with children** | | | | | | |
| | n | %[a] | n | %[b] | n | %[b] | n | %[b] | |
| **Overall** | 529 | 16.7 | 303 | 57.5 | 138 | 26.6 | 88 | 15.9 | <0.001 |
| **Sex** | | | | | | | | | 0.143 |
| Men | 316 | 58.4 | 170 | 55.5 | 89 | 27.3 | 57 | 17.2 | |
| Women | 213 | 41.6 | 133 | 60.3 | 49 | 25.6 | 31 | 14.1 | |
| **Age (years)** | | | | | | | | | 0.014 |
| 18−24 | 47 | 14.3 | 24 | 53.0 | 9 | 19.6 | 14 | 27.4 | |
| 25−39 | 170 | 38.3 | 87 | 52.5 | 50 | 30.0 | 33 | 17.5 | |
| 40−54 | 188 | 30.8 | 109 | 57.3 | 55 | 30.6 | 24 | 12.1 | |
| ≥ 55 | 124 | 16.6 | 83 | 73.0 | 24 | 14.5 | 17 | 12.5 | |
| **Educational level** | | | | | | | | | 0.431 |
| Low | 81 | 24.8 | 44 | 56.1 | 24 | 27.5 | 13 | 16.4 | |
| Intermediate | 353 | 63.0 | 202 | 56.7 | 86 | 26.0 | 65 | 17.3 | |
| High | 85 | 12.2 | 52 | 61.7 | 24 | 27.4 | 9 | 10.9 | |
| **Smoker partner** | | | | | | | | | 0.567 |
| Yes | 202 | 57.7 | 112 | 55.7 | 59 | 28.9 | 31 | 15.4 | |
| No | 195 | 42.3 | 117 | 60.8 | 48 | 24.4 | 30 | 14.8 | |
| **Children** | | | | | | | | | 0.169 |
| Yes | 180 | 38.9 | 102 | 56.9 | 41 | 24.1 | 37 | 19.0 | |
| No | 348 | 61.1 | 201 | 58.0 | 96 | 27.9 | 51 | 14.1 | |
| **Number of children** | | | | | | | | | 0.104 |
| 0 | 348 | 61.2 | 201 | 58.0 | 96 | 27.9 | 51 | 14.1 | |
| 1 | 110 | 19.7 | 61 | 56.2 | 23 | 22.2 | 26 | 21.6 | |
| 2 | 62 | 14.9 | 36 | 59.0 | 18 | 30.4 | 8 | 10.6 | |
| ≥3 | 8 | 4.2 | 5 | 49.0 | 0 | - | 3 | 51.0 | |
| **Children's age (years)[d]** | | | | | | | | | |
| <1 | 12 | 7.6 | 9 | 69.8 | 0 | - | 3 | 30.2 | 0.148 |
| 1−5 | 69 | 43.8 | 42 | 61.2 | 15 | 23.1 | 12 | 15.7 | 0.623 |
| 6−12 | 91 | 54.3 | 53 | 59.1 | 24 | 27.5 | 14 | 13.4 | 0.172 |
| 13−17 | 56 | 36.5 | 28 | 48.6 | 14 | 26.5 | 14 | 24.9 | 0.450 |
| **Cigarettes smoked per day** | | | | | | | | | 0.001 |
| ≤10 | 188 | 32.5 | 128 | 69.4 | 40 | 18.9 | 20 | 11.7 | |
| 11−20 | 277 | 55.9 | 148 | 52.5 | 81 | 32.1 | 48 | 15.4 | |
| 21−30 | 38 | 6.9 | 17 | 52.8 | 11 | 22.5 | 10 | 24.7 | |
| >30 | 26 | 4.7 | 10 | 38.5 | 6 | 19.6 | 10 | 41.9 | |
| **Nicotine dependence** | | | | | | | | | 0.078 |
| Low | 206 | 34.5 | 127 | 62.4 | 49 | 21.6 | 30 | 16.0 | |
| Medium | 248 | 56.6 | 129 | 52.1 | 74 | 32.4 | 45 | 15.5 | |
| High | 46 | 8.9 | 21 | 48.0 | 12 | 23.1 | 13 | 28.9 | |
| **Attempts to quit smoking** | | | | | | | | | 0.033 |
| Yes | 320 | 62.4 | 197 | 62.1 | 72 | 21.7 | 51 | 16.2 | |
| No | 209 | 37.6 | 106 | 50.0 | 66 | 34.4 | 37 | 15.6 | |

[a] Weighted percentages per column.
[b] Weighted percentages per row.
[c] $\chi^2$ test.
[d] Multiple response.

**Country: Spain**

| | Overall | | Smoking rules in cars with children: Total ban | | Partial rules | | No rules | | p[c] |
|---|---|---|---|---|---|---|---|---|---|
| | n | %[a] | n | %[b] | n | %[b] | n | %[b] | |
| **Overall** | 693 | 16.7 | 436 | 61.5 | 114 | 18.1 | 143 | 20.4 | <0.001 |
| **Sex** | | | | | | | | | 0.634 |
| Men | 373 | 57.3 | 235 | 61.4 | 65 | 18.7 | 73 | 19.9 | |
| Women | 320 | 42.7 | 201 | 61.7 | 49 | 17.4 | 70 | 20.9 | |
| **Age (years)** | | | | | | | | | 0.305 |
| 18−24 | 71 | 12.1 | 38 | 44.3 | 18 | 33.4 | 15 | 22.3 | |
| 25−39 | 220 | 29.0 | 137 | 63.8 | 35 | 15.4 | 48 | 20.8 | |
| 40−54 | 241 | 38.5 | 151 | 61.7 | 41 | 18.3 | 49 | 20.0 | |
| ≥ 55 | 161 | 20.4 | 110 | 67.5 | 20 | 13.2 | 31 | 19.3 | |
| **Educational level** | | | | | | | | | 0.579 |
| Low | 268 | 44.2 | 169 | 59.4 | 38 | 16.4 | 61 | 24.2 | |
| Intermediate | 361 | 47.9 | 229 | 63.9 | 64 | 18.5 | 68 | 17.6 | |
| High | 63 | 7.9 | 37 | 57.8 | 12 | 24.8 | 14 | 17.4 | |
| **Smoker partner** | | | | | | | | | 0.005 |
| Yes | 276 | 57.6 | 161 | 56.4 | 49 | 19.0 | 66 | 24.6 | |
| No | 190 | 42.4 | 138 | 71.1 | 26 | 13.1 | 26 | 15.8 | |
| **Children** | | | | | | | | | 0.938 |
| Yes | 284 | 35.1 | 180 | 65.0 | 45 | 15.3 | 59 | 19.7 | |
| No | 409 | 64.9 | 256 | 58.9 | 69 | 20.3 | 84 | 20.8 | |
| **Number of children** | | | | | | | | | 0.038 |
| 0 | 409 | 64.9 | 256 | 58.9 | 69 | 20.3 | 84 | 20.8 | |
| 1 | 170 | 22.1 | 108 | 63.4 | 23 | 12.8 | 39 | 23.8 | |
| 2 | 89 | 10.3 | 63 | 72.4 | 13 | 15.0 | 13 | 12.6 | |
| ≥3 | 25 | 2.7 | 9 | 47.8 | 9 | 35.0 | 7 | 17.2 | |
| **Children's age (years)[d]** | | | | | | | | | |
| <1 | 16 | 5.9 | 9 | 36.7 | 2 | 25.0 | 5 | 38.3 | 0.562 |
| 1−5 | 101 | 33.7 | 62 | 62.7 | 17 | 16.0 | 22 | 21.3 | 0.873 |
| 6−12 | 145 | 47.6 | 91 | 66.0 | 32 | 21.3 | 22 | 12.7 | 0.003 |
| 13−17 | 103 | 38.4 | 65 | 66.8 | 14 | 13.1 | 24 | 20.1 | 0.602 |
| **Cigarettes smoked per day** | | | | | | | | | <0.001 |
| ≤10 | 306 | 40.1 | 227 | 72.6 | 36 | 14.1 | 43 | 13.3 | |
| 11−20 | 320 | 48.1 | 180 | 53.7 | 64 | 21.6 | 76 | 24.7 | |
| 21−30 | 40 | 7.2 | 17 | 46.7 | 9 | 23.1 | 14 | 30.2 | |
| >30 | 27 | 4.6 | 12 | 59.0 | 5 | 14.0 | 10 | 27.0 | |
| **Nicotine dependence** | | | | | | | | | <0.001 |
| Low | 376 | 50.0 | 261 | 67.6 | 59 | 18.4 | 56 | 14.0 | |
| Medium | 242 | 41.6 | 138 | 55.6 | 41 | 18.0 | 63 | 26.4 | |
| High | 49 | 8.4 | 17 | 45.8 | 12 | 21.1 | 20 | 33.1 | |
| **Attempts to quit smoking** | | | | | | | | | 0.974 |
| Yes | 451 | 61.7 | 285 | 60.7 | 74 | 18.3 | 92 | 21.0 | |
| No | 242 | 38.3 | 151 | 63.0 | 40 | 17.8 | 51 | 19.2 | |

[a] Weighted percentages per column.
[b] Weighted percentages per row.
[c] $\chi^2$ test.
[d] Multiple response.

© 2019 Fu M.
